# Longitudinal cognitive biomarkers predicting symptom onset in presymptomatic frontotemporal dementia

**DOI:** 10.1007/s00415-018-8850-7

**Published:** 2018-04-07

**Authors:** Lize C. Jiskoot, Jessica L. Panman, Lauren van Asseldonk, Sanne Franzen, Lieke H. H. Meeter, Laura Donker Kaat, Emma L. van der Ende, Elise G. P. Dopper, Reinier Timman, Rick van Minkelen, John C. van Swieten, Esther van den Berg, Janne M. Papma

**Affiliations:** 1 Department of Neurology, Erasmus Medical Center Rotterdam, Room Ee2240, ’s-Gravendijkwal 230, 3015 CE Rotterdam, The Netherlands; 2 Department of Radiology, Leiden University Medical Center, Leiden, The Netherlands; 3 Department of Clinical Genetics, Leiden University Medical Center, Leiden, The Netherlands; 4 Department of Psychiatry, Section of Medical Psychology and Psychotherapy, Erasmus Medical Center, Rotterdam, The Netherlands; 5 Department of Clinical Genetics, Erasmus Medical Center, Rotterdam, The Netherlands; 6 Department of Clinical Genetics, VU Medical Center, Amsterdam, The Netherlands

**Keywords:** Presymptomatic, Frontotemporal dementia, Familial, Biomarkers, Cognition, Neuropsychological assessment, Longitudinal

## Abstract

**Introduction:**

We performed 4-year follow-up neuropsychological assessment to investigate cognitive decline and the prognostic abilities from presymptomatic to symptomatic familial frontotemporal dementia (FTD).

**Methods:**

Presymptomatic *MAPT* (*n *= 15) and *GRN* mutation carriers (*n *= 31), and healthy controls (*n *= 39) underwent neuropsychological assessment every 2 years. Eight mutation carriers (5 *MAPT*, 3 *GRN*) became symptomatic. We investigated cognitive decline with multilevel regression modeling; the prognostic performance was assessed with ROC analyses and stepwise logistic regression.

**Results:**

*MAPT* converters declined on language, attention, executive function, social cognition, and memory, and *GRN* converters declined on attention and executive function (*p *< 0.05). Cognitive decline in ScreeLing phonology (*p *= 0.046) and letter fluency (*p *= 0.046) were predictive for conversion to non-fluent variant PPA, and decline on categorical fluency (*p *= 0.025) for an underlying *MAPT* mutation.

**Discussion:**

Using longitudinal neuropsychological assessment, we detected a mutation-specific pattern of cognitive decline, potentially suggesting prognostic value of neuropsychological trajectories in conversion to symptomatic FTD.

**Electronic supplementary material:**

The online version of this article (10.1007/s00415-018-8850-7) contains supplementary material, which is available to authorized users.

## Introduction

Frontotemporal dementia (FTD) is a presenile neurodegenerative disorder, leading to a heterogeneous clinical presentation, involving behavioural (behavioural variant FTD; bvFTD) and/or language deterioration (primary progressive aphasia; PPA) [1]. FTD has an autosomal dominant pattern of inheritance in 30 percent of cases, with mutations in the progranulin (*GRN*) and microtubule-associated protein tau (*MAPT*) genes as its two main causes [2]. The cognitive profile of FTD varies depending on the clinical phenotype and the underlying genotype. Patients with bvFTD are characterized by deficits in executive function, social cognition and language, whereas memory and visuoconstruction are initially spared [3–5]. Non-fluent variant PPA (nfvPPA) patients show agrammatism and speech sound distortions, while semantic variant PPA (svPPA) patients experience deficits in confrontation naming and word comprehension [6]. *GRN* mutations often lead to a clinical diagnosis of bvFTD, nfvPPA or parkinsonism. In *MAPT* mutations, bvFTD is the main phenotype, and semantic and memory impairments can be prominent neuropsychological symptoms [7].

Research in familial FTD has demonstrated the presence of a presymptomatic stage in which subtle cognitive changes have been identified [8–12]. More specifically, cognitive decline can start as early as 8 years prior to estimated symptom onset and shows mutation-specific patterns, with *GRN* mutation carriers declining in memory, and *MAPT* mutation carriers declining in language, social cognition and memory [8, 10]. This suggests that cognitive measures could function as disease-tracking biomarkers in the presymptomatic stage. However, it is currently unknown what the long-term cognitive profiles of presymptomatic FTD mutations are, whether neuropsychological assessment can be used to track disease progression to the symptomatic stage, and what the prognostic value is of cognitive trajectories in the presymptomatic and early symptomatic stage of FTD.

In this study, we investigated longitudinal cognitive decline on neuropsychological assessment in presymptomatic mutation carriers (*MAPT* or *GRN*) and controls from the same families within our longitudinal presymptomatic Dutch familial FTD Risk Cohort (FTD-RisC). Second, we assessed the difference in cognitive course between converters’ genotypes (i.e. *MAPT* vs. *GRN*) and phenotypes (i.e. bvFTD vs. nfvPPA) versus non-converters. Lastly, we investigated the prognostic value of neuropsychological trajectories in predicting symptom onset within 2–4 years.

## Methods

### Participants

**Fig. 1:**
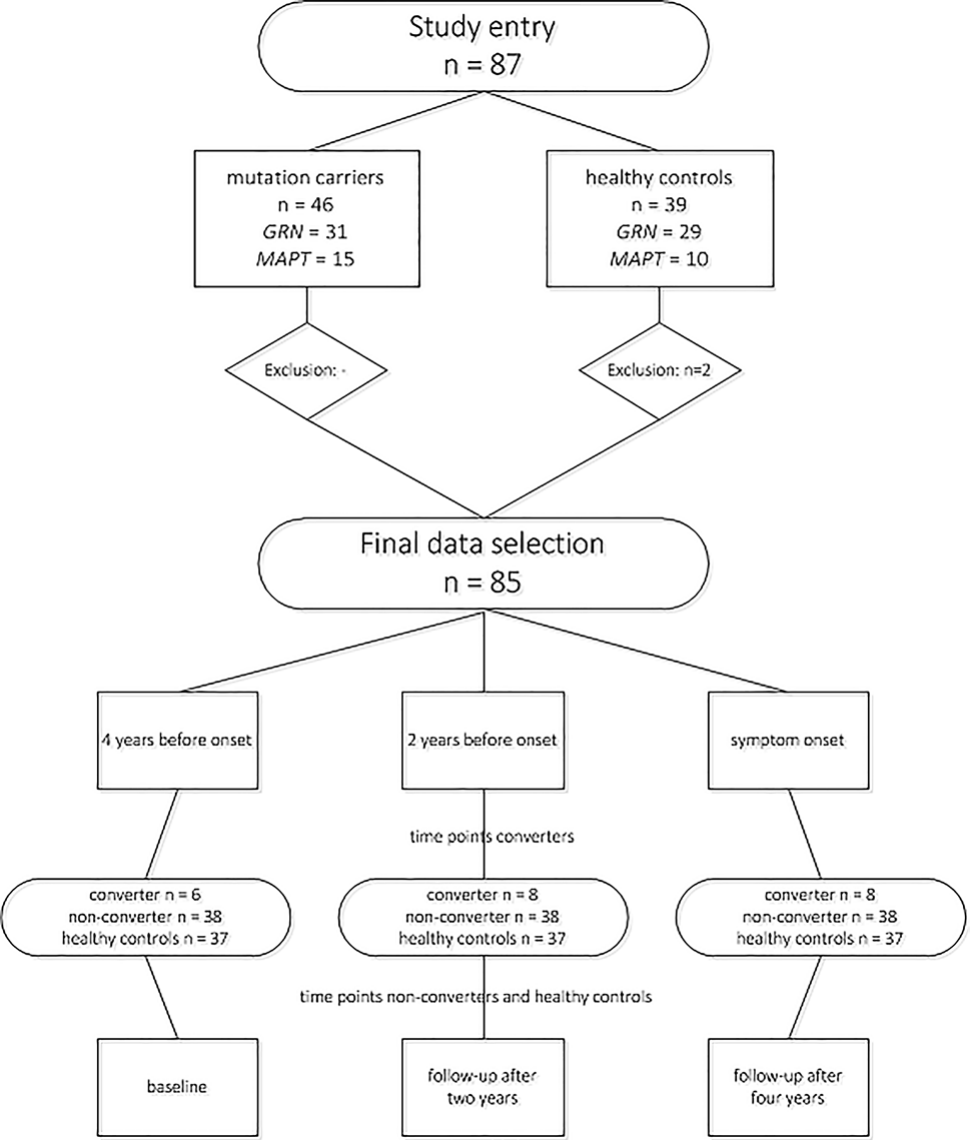
Participant in- and exclusion and sample size per time point. Two controls were excluded as they had multiple cognitive disorders (≤ 2 SD below reference mean) on neuropsychological testing. Eight mutation carriers converted to clinical FTD within the study window. Their data were restructured, so that there were three time points: 4 years before symptom onset, 2 years before symptom onset and symptom onset. Four years before symptom onset, only data of six converters were available, as two mutation carriers converted between baseline and first follow-up. The data of converters were compared to, respectively, baseline, follow-up after 2 years and follow-up after 4 years in non-converters and healthy controls

In FTD-RisC, we follow healthy 50% at-risk family members from genetic FTD families on a 2-year basis. In the current study, we included 87 participants from *MAPT* or *GRN* families with study entries between December 2009 and January 2013 [8, 9, 13]. The follow-up period was 4 years, in which we acquired neuropsychological assessments at study entry, follow-up after 2 years and follow-up after 4 years. DNA genotyping (see “Procedure”) assigned participants either to the presymptomatic mutation carrier (*n* = 46; 31 *GRN*, 15 *MAPT*), or control group (*n* = 39; 29 *GRN*, 10 *MAPT* family members). We excluded two controls as they had cognitive disorders (≥ 2 SD below mean) on multiple domains, ultimately including 85 participants (46 mutation carriers, 37 controls; Fig. 1).

### Standard protocol approvals, registrations, and patient consents

Clinical investigators were blind for participants’ genetic status if they had not undergone predictive testing. In case of conversion to clinical FTD, we offered the patient and family members genetic counselling and unblinding of genetic status, to confirm the presence of the pathogenic mutation. At study entry, all participants gave written informed consent. The study was approved by the Medical and Ethical Review Committee of the Erasmus Medical Center.

### Procedure

Every 2 years, participants underwent a standardized assessment consisting of a neuropsychological test battery, neurological examination, and MR imaging of the brain. DNA sequencing was performed at study entry. All participants were asymptomatic according to established diagnostic criteria for bvFTD [3] or PPA [6] at baseline. Knowledgeable informants were asked about cognitive and/or behavioural deterioration at each study visit by means of a structured interview and a well-validated questionnaire (Neuropsychiatric Inventory; NPI) [14].

### Converters

Eight mutation carriers became symptomatic within the study time window (“converters”). Symptom onset was determined by means of the above mentioned assessment (anamnesis, MR imaging of the brain, neuropsychological assessment, heteroanamnestic information and unblinding of genetic status). Conversion was determined in a multidisciplinary consensus meeting of the Erasmus MC FTD Expertise Centre, involving neurologists (LDK, JCvsS), neuropsychologists (LCJ, JLP, SF, EvdB, JMP), medical doctors (LHM, ELvdE), as well as neuroradiologists, geriatricians, a clinical geneticist (RvM), and a care consultant. Six converters (5 *MAPT*, 1 *GRN*) presented with progressive behaviour deterioration, functional decline, and frontal and/or temporal lobe atrophy on MRI, fulfilling the international diagnostic consensus criteria of Rascovsky et al. [3] for bvFTD with definite FTLD pathology. Two converters (both *GRN*) presented with isolated language difficulties and no impairments in daily living activities, thereby fulfilling the diagnostic criteria for PPA of Gorno-Tempini et al. [6]. Both developed nfvPPA, as they showed a non-fluent, halting speech, with sound errors and agrammatism. See Supplementary Table 1 for demographic, clinical and neuropsychological data of the converters. We defined mutation carriers remaining without FTD symptoms as non-converters (*n* = 38; 28 *GRN*, 10 *MAPT*).

### Neuropsychological assessment

We screened global cognitive functioning by means of the Mini-Mental State Examination [15] (MMSE) and frontal assessment battery [16] (FAB). Experienced neuropsychologists (LCJ, JLP, SF) administered neuropsychological tests within six cognitive domains: language, attention and mental processing speed, executive functioning, social cognition, memory, and visuoconstruction. We rated language with the 60-item Boston Naming Test (BNT) [17], verbal Semantic Association Test (SAT) [18], ScreeLing phonology [19], and categorical fluency [20]. We assessed attention and mental processing speed by means of Trail making Test (TMT)-A [21], Stroop Color-Word Test I and II [22], Wechsler Adult Intelligence Scale III (WAIS-III) Digit Span forwards [23], and Letter Digit Substitution Test (LDST) [24]. Executive functioning was evaluated using TMT-B [21], Stroop Color-Word Test III [22], WAIS-III Digit Span backwards [23], modified Wisconsin Card Sorting Test (WCST) concepts [25], letter fluency [20], and WAIS-III Similarities [23]. Happé cartoons [26] and Ekman Faces [27] measured social cognition. We assessed memory using the Dutch Rey Auditory Verbal Learning Test (RAVLT) [28] and Visual Association Test (VAT) [29]. We evaluated visuoconstruction by means of clock drawing [30] and WAIS-III Block Design [23]. Alternate forms were used at follow-up visits, when applicable (letter fluency, RAVLT, VAT). Depressive symptoms were rated with the Beck’s Depression Inventory (BDI) [31].

### Study design

In converters, we restructured the three original time points within our study window (i.e. baseline, follow-up after 2 years, follow-up after 4 years) into the following three new time points (Fig. 1):

- 4 years before symptom onset: we used the data of the study visit 4 years before diagnosis. Analyses could were performed in six converters, as two (1 *GRN*, 1 *MAPT*—2 bvFTD) developed symptoms between baseline and first follow-up (i.e. at 2 years follow-up), and therefore no data 4 years prior to symptom onset were available.
- 2 years before symptom onset: we used the data of the study visit 2 years before diagnosis. Analyses included all eight converters.
- After symptom onset: we used the data of the diagnosis visit. Analyses included all eight converters.

In non-converters and controls, we used the original time points: baseline (data were compared to “4 years before symptom onset” data of converters), follow-up after 2 years (data were compared to “2 years before symptom onset data of converters) and follow-up after 4 years (data were compared to “after symptom onset data of converters).

### Statistical analysis

Statistical analyses were performed using SPSS Statistics 21.0 (IBM Corp., Armonk, NY) and GraphPad Prism 7 (La Jolla, California, USA), with the significance level at *p *< 0.05 (two-tailed) across all comparisons. We compared demographic data between *MAPT* mutation carriers, *GRN* mutation carriers and controls, and between converters, non-converters and controls by means of one-way ANOVAs. We performed Pearson *Χ*^2^ tests to investigate differences in sex. Longitudinal comparisons of clinical data were performed with repeated measures ANOVAs. We standardized all raw neuropsychological test scores by converting them into *z*-scores (i.e. individual test score minus the baseline mean of the controls, divided by the baseline SD of the controls) per time point, after which we calculated composite *z*-scores for the respective six cognitive domains by averaging the *z*-scores of the individual tests per domain. For the longitudinal comparisons we used multilevel linear regression modeling. This analysis corrects for bias when data absence is dependent on characteristics present in the model, and can therefore efficiently handle missing and unbalanced time points. There were two levels in the models: the participants constituted the upper level; their repeated measures the lower level. We ran two analyses to assess cognitive decline per mutation (1) and clinical status (2):

1. We entered mutation status (*MAPT* mutation carrier, *GRN* mutation carrier or control), time (4 years before symptom onset, 2 years before symptom onset, and after symptom onset), and first-order interactions, with age, gender and educational level as covariates. We reran the analyses excluding the converters to exclude converters driving the cognitive decline in the mutation carrier groups;
2. We split the converter group according to genotype (*MAPT* or *GRN*) and phenotype (bvFTD or nfvPPA) to investigate specific profiles of cognitive decline over time. We then entered clinical status (converter, non-converter or control), time, and first-order interactions, with age, gender and educational level as covariates.

Third, to investigate the prognostic abilities of cognitive decline in discriminating between converters and non-converters, we determined the area under the curve (AUC) by receiver operating characteristic (ROC) analyses on the neuropsychological trajectories between visits. For this, we calculated deltas between test scores; one between 4 and 2 years before symptom onset and one between 2 years before symptom onset and symptom onset. Optimal cut-off levels were given by the highest Youden’s index [32]. Again, we split the converter group according to genotype (*MAPT* or *GRN*) and phenotype (bvFTD or nfvPPA). Next, we performed logistic regression analyses, taking group (converter vs. non-converter) as the dependent variable and the deltas (tests with significant diagnostic performance in abovementioned ROC analyses) as the independent variables. The models were selected with a forward stepwise method according to the likelihood ratio test and applying the standard *p* values for variable inclusion (0.05) and exclusion (0.10), with age, sex and education as covariates. Goodness of fit was evaluated with the HL *Χ*^2^ test. Nagelkerke *R*^2^ is reported as measure of effect size. We checked predictor variables for multicollinearity. All models were corrected for multiple comparisons (Bonferroni).

## Results

### Demographics

*MAPT* mutation carriers were significantly younger than *GRN* mutation carriers (*p *= 0.012; Table 1). The mean familial symptom onset age was lower in *MAPT* than in *GRN* mutation carriers and controls (both *p *< 0.001). There were no significant differences between groups regarding estimated years to symptom onset (*p *> 0.05). Longitudinal analyses demonstrated that *MAPT* mutation carriers declined significantly more than *GRN* mutation carriers and controls with regards to the MMSE (*p *= 0.014), and also developed more depressive symptoms (*p *= 0.028). FAB and NPI scores did not significantly change over time (*p *> 0.05). Converters, non-converters and controls did not differ regarding demographic variables, apart from a younger family onset in *MAPT* converters than *GRN* converters (*p *= 0.043) and non-converters (*p *= 0.001; Table 1). Both *MAPT* and *GRN* converters declined significantly with respect to MMSE score (*p *< 0.001) and they developed more neuropsychiatric symptoms in the form of higher BDI (*p *= 0.001) and NPI (*p *= 0.021) scores in comparison to non-converters and controls. FAB scores did not significantly change over time (*p *> 0.05).

**Table 1:** Demographics and clinical data

Demographics		HC (*n* = 39)	*MAPT* carriers (*n* = 15)	*GRN* carriers (*n* = 31)	*p* value*	*MAPT* converters (*n* = 5)	*GRN* converters (*n* = 3)	Non-converters (*n* = 38)	*p* value**
Age at study entry, years		49.1 ± 12.2	41.9 ± 10.0	52.1 ± 8.2	**0.012** ^a^	45.3 ± 8.5	54.9 ± 9.0	48.8 ± 10.3	0.704
Sex, female (%)		20 (56%)	7 (47%)	20 (65%)	0.506	1 (20%)	3 (100%)	23 (60.5%)	0.154
Education (Verhage)^f^		5.2 ± 1.0	5.1 ± 1.6	5.7 ± 0.9	0.102	6.0 ± 0.7	5.7 ± 0.6	5.4 ± 1.3	0.409
Onset age family, years		59.0 ± 5.8	51.3 ± 6.7	61.0 ± 2.4	**<** **0.001**^a,b^	48.0 ± 4.7	59.7 ± 0.0	58.8 ± 6.1	**0.002** ^c,d^
Estimated years to onset, years		− 10.2 ± 11.2	− 7.7 ± 9.6	− 9.4 ± 7.9	0.690	− 2.7 ± 4.0	− 4.8 ± 9.0	− 10.0 ± 8.5	0.335

Clinical data	Years to onset	HC (*n* = 39)	*MAPT* carriers (*n* = 15)	*GRN* carriers (*n* = 31)	*p* value*	*MAPT* converters (*n* = 5)	*GRN* converters (*n* = 3)	Non-converters (*n* = 38)	*p* value**
MMSE	4	29.1 ± 1.3	29.6 ± 0.5	29.1 ± 1.6	0.451	29.5 ± 0.6	29.0 ± 1.4	29.2 ± 1.4	0.924
	2	29.2 ± 1.3	28.7 ± 2.2	28.9 ± 1.6	0.513	29.8 ± 0.4	28.0 ± 1.0	27.7 ± 1.5	0.271
	0	29.2 ± 1.0	28.4 ± 1.5	29.2 ± 1.4	0.099	27.2 ± 1.6	27.7 ± 1.5	29.3 ± 1.2	**0.001** ^c,d^
FAB^g^	4	–	–	–	–	–	–	–	–
	2	17.4 ± 0.9	17.4 ± 0.8	17.5 ± 0.9	0.883	17.3 ± 1.0	17.5 ± 0.7	17.5 ± 0.9	0.929
	0	16.7 ± 1.7	16.5 ± 1.6	17.0 ± 1.1	0.639	15.4 ± 1.5	16.3 ± 1.5	17.1 ± 1.1	0.120
BDI	4	4.1 ± 4.5	4.0 ± 6.3	3.2 ± 3.9	0.693	1.3 ± 1.0	2.0 ± 2.8	3.7 ± 5.0	0.645
	2	3.7 ± 3.9	4.5 ± 5.0	3.2 ± 4.0	0.638	5.0 ± 4.7	2.7 ± 3.8	3.5 ± 4.4	0.866
	0	3.5 ± 4.3	7.6 ± 9.5	3.0 ± 6.7	0.108	11.6 ± 13.0	6.3 ± 5.1	3.1 ± 6.5	**0.042** ^c,d^
NPI	4	0.1 ± 0.5	4.6 ± 11.2	1.4 ± 3.4	0.180	0.0 ± 0.0	–	3.0 ± 7.5	**0.006** ^c–e^
	2	0.6 ± 1.2	6.4 ± 20.7	0.3 ± 0.7	0.095	0.2 ± 0.4	0.0 ± 0.0	2.9 ± 13.3	0.767
	0	0.8 ± 1.5	12.3 ± 18.7	2.1 ± 6.6	**0.001** ^a,b^	15.6 ± 16.3	10.7 ± 15.9	3.4 ± 11.4	**0.009** ^d^

Values indicate: mean ± standard deviation. Significant comparisons are displayed in bold

*GRN* progranulin, *HC* healthy control, *MMSE* Mini-Mental State Examination, *FAB* frontal assessment battery, *BDI* Beck’s depression inventory, *NPI* neuropsychiatric inventory

*p value represents result of overall ANOVA between *MAPT* mutation carriers, *GRN* mutation carriers and healthy controls

**p value represents result of overall ANOVA between* MAPT* converters,* GRN* converters, non-converters and HC

^a^Significant post hoc test between* MAPT* and* GRN* mutation carriers

^b^Significant post hoc test between* MAPT* mutation carriers and healthy controls

^c^Significant post hoc test between converters and non-converters

^d^Significant post hoc test between converters and healthy controls

^e^Only data of* MAPT* converters available, therefore the *p* value represents the comparison between* MAPT* converters, non-converters and HC

^f^Dutch educational system categorized into levels from 1 = less than 6 years of primary education to 7 = academic schooling (Verhage, 1964)

^g^Data only available on follow-up visits

### Longitudinal cognitive decline in *MAPT* and *GRN* mutation carriers

The whole group of *MAPT* mutation carriers declined significantly within the domains language, social cognition and memory compared with controls (Table 2; Fig. 1). This was reflected in lower scores on the BNT and categorical fluency, Happé cartoons, VAT and RAVLT delayed recall (Table 2). In the whole group of *GRN* mutation carriers, no longitudinal decline was found in comparison to controls. In comparison to *GRN* mutation carriers, *MAPT* mutation carriers declined significantly on the domains language (*β* = − 0.015, *p *< 0.001) and memory (*β* = − 0.016, *p *= 0.008), reflected in lower BNT (*β* = − 0.085, *p *= 0.01), SAT (*β* = − 0.027, *p *= 0.015), category fluency (*β* = − 0.107, *p *= 0.002), and RAVLT delayed recall (*β* = − 0.047, *p *= 0.001) scores. There were no cognitive domains or tests on which *GRN* mutation carriers declined more than *MAPT* mutation carriers (Table 2). By excluding the five *MAPT* converters from the analyses, none of the domain scores in *MAPT* mutation carriers continued to show significant decline over time in comparison to controls. Regarding individual tests, however, the decline on the RAVLT delayed recall remained significant (*β* = − 0.032, *p *= 0.023). The results did not change by excluding the three *GRN* converters from the analyses. In comparison to *GRN, MAPT* mutation carriers still declined more on language (*β* = − 0.010, *p *= 0.004), reflected in lower ScreeLing phonology (*β* = − 0.008, *p *= 0.024) and category fluency (*β* = − 0.007, *p *= 0.041). There was no cognitive decline in controls—but significant improvement was found on social cognition (Happé non-ToM and Ekman Faces) and memory (RAVLT immediate and delayed recall) (Table 2). The raw neuropsychological test scores per time point can be found in Supplementary Table 2.

**Table 2:** Cognitive trajectories in mutation carriers (converters, non-converters) and healthy controls

Domain test	Healthy controls (*n* = 39)			*MAPT* mutation carriers (*n* = 15)			*GRN* mutation carriers (*n* = 31)		
	Baseline	*β*	*p*	Baseline	*β*	*p*	Baseline	*β*	*p*
Language	0.0 ± 0.6	0.000	0.931	0.2 ± 0.6	− 0.010	**0.002**	0.1 ± 0.7	0.004	0.121
BNT	53.4 ± 4.5	0.026	0.105	52.6 ± 5.3	− 0.080	**0.005**	55.1 ± 3.7	0.006	0.786
SAT	27.8 ± 1.1	− 0.003	0.604	27.9 ± 1.5	− 0.008	0.604	27.5 ± 2.0	0.019	**0.033** ^a^
ScreeLing phonology	23.5 ± 0.8	0.001	0.733	23.9 ± 0.3	− 0.005	0.190	23.8 ± 0.5	− 0.001	0.863
Categorical fluency	23.9 ± 4.9	0.026	0.141	26.5 ± 6.6	− 0.087	**0.006**	23.4 ± 5.7	0.021	0.424
Attention and processing speed	0.0 ± 0.8	− 0.001	0.084	0.3 ± 0.6	− 0.003	0.096	0.1 ± 0.9	− 0.003	0.075
TMT part A^c^	31.8 ± 15.0	− 0.022	0.416	26.1 ± 9.7	0.065	0.192	31.4 ± 12.2	0.060	0.145
Stroop card I^c^	47.1 ± 8.0	0.039	**0.011**	43.2 ± 8.8	− 0.017	0.529	45.0 ± 8.4	− 0.001	0.951
Stroop card II^c^	58.5 ± 10.6	0.012	0.539	54.9 ± 8.5	0.027	0.470	60.2 ± 13.2	0.001	0.969
Digit Span forwards	8.7 ± 1.9	0.001	0.871	9.0 ± 2.6	− 0.010	0.294	9.4 ± 2.4	− 0.016	0.055
LDST	34.5 ± 6.8	0.001	0.894	34.2 ± 4.7	− 0.636	0.699	33.2 ± 7.4	0.005	0.798
Executive function	0.0 ± 0.7	0.001	0.505	0.3 ± 0.6	− 0.005	0.065	0.2 ± 0.8	− 0.004	0.052
TMT part B^c^	67.8 ± 29.3	0.052	0.494	61.0 ± 28.5	0.079	0.570	72.2 ± 42.7	− 0.099	0.390
Stroop card III^c^	93.7 ± 22.6	− 0.087	**0.021**	83.8 ± 14.7	0.141	**0.042**	96.6 ± 26.2	0.013	0.815
Digit span backwards	6.1 ± 2.0	0.008	0.194	6.6 ± 1.8	0.002	0.877	6.6 ± 2.1	− 0.011	0.222
WCST concepts	5.5 ± 0.9	0.002	0.592	5.6 ± 1.1	− 0.009	0.296	5.80 ± 0.6	− 0.010	0.144
Letter fluency	32.1 ± 9.9	0.134	**<** **0.001**^b^	36.1 ± 14.3	− 0.108	**0.049**	38.9 ± 12.0	− 0.062	0.173
Similarities	24.8 ± 4.7	0.006	0.645	25.5 ± 4.7	− 0.034	0.122	26.2 ± 5.0	− 0.011	0.556
Social cognition	0.0 ± 0.8	0.000	0.878	0.2 ± 0.7	− 0.009	**0.007**	0.3 ± 0.7	− 0.003	0.332
Happé ToM	11.8 ± 3.4	0.013	0.172	12.6 ± 3.7	− 0.044	**0.011**	12.9 ± 2.9	− 0.005	0.707
Happé non-Tom	11.7 ± 2.9	0.020	**0.013**	12.4 ± 2.8	− 0.036	**0.017**	13.0 ± 2.6	− 0.012	0.331
Ekman faces	45.7 ± 6.4	0.038	**0.009**	47.0 ± 5.5	− 0.028	0.293	47.10 ± 5.5	− 0.013	0.548
Memory	0.0 ± 0.7	0.000	0.848	0.1 ± 1.3	− 0.017	**<** **0.001**^b^	0.1 ± 0.9	− 0.001	0.745
VAT	11.8 ± 0.6	0.001	0.740	11.4 ± 1.6	− 0.012	**0.019**	11.5 ± 0.9	0.000	0.926
RAVLT imm. recall	42.6 ± 9.8	0.157	**<** **0.001**^b^	47.5 ± 9.7	− 0.076	0.090	46.3 ± 10.6	− 0.015	0.686
RAVLT del. recall	8.4 ± 3.2	0.050	**<** **0.001**^b^	9.7 ± 3.9	− 0.048	**<** **0.001**^a,b^	9.4 ± 3.3	− 0.000	0.983
RAVLT recognition	28.6 ± 2.1	0.014	0.127	29.0 ± 2.0	− 0.022	0.176	29.2 ± 1.2	− 0.009	0.505
Visuoconstruction	0.0 ± 0.8	− 0.001	0.656	− 0.2 ± 0.7	− 0.005	0.266	0.0 ± 1.0	0.000	0.963
Block design	36.5 ± 14.0	0.034	0.305	35.5 ± 20.8	− 0.006	0.917	39.3 ± 18.5	− 1.164	0.246
Clock drawing	12.6 ± 1.4	0.003	0.453	12.2 ± 1.3	− 0.009	0.284	12.4 ± 1.8	0.005	0.475

Values indicate: mean ± standard deviation; *β* represents estimate of change over time. Composite domain scores are *z*-scores, individual test scores are raw scores. Composite domain scores are expressed as *z*-scores, the individual test scores are raw scores. *p* values represent comparisons to healthy controls. Significant comparisons are displayed in bold

*MAPT* microtubule-associated protein tau, *GRN* progranulin, *BNT* Boston Naming Test, *SAT* semantic association test, *TMT* Trail making Test, *WAIS* Wechsler Adult Intelligence Scale, *LDST* letter digit substitution test, *WCST* Wisconsin card sorting test, *ToM* theory of mind, *VAT* visual association test, *RAVLT* Rey Auditory Verbal Learning Test, *imm* immediate, *del* delayed

^a^Remained significant after excluding converters from the analyses

^b^Survived Bonferroni correction for multiple comparisons

^c^Higher scores and *β* weights indicate worse performance

### Longitudinal cognitive decline in converters and non-converters

**Fig. 2:**
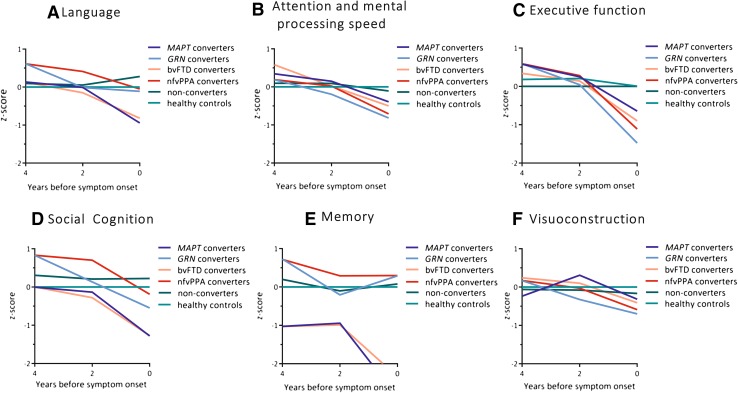
Multilevel linear regression model displaying longitudinal decline (4 years, 2 years and after symptom onset) in composite domain *z*-score in the total group of converters (light green), *MAPT* converters (light blue dotted line), *GRN* converters (dark blue dotted line), non-converters (dark green) and healthy controls (black). Models are displayed per cognitive domain: **a** social cognition, **b** attention and mental processing speed, **c** executive functioning, **d** memory, **e** visuoconstruction, and **f** language. NB: the healthy controls have a mean *z*-score of zero by default as the *z*-scores of mutation carriers were based on that (raw score minus mean score of healthy controls, divided by the standard deviation of healthy controls). *MAPT* microtubule-associated protein tau, *GRN* progranulin

Converters with a *MAPT* mutation deteriorated significantly on all domains but visuoconstruction (Fig. 2a–d, f; Table 3). Within these domains, performances declined on BNT (*p *< 0.001), LDST (*p *= 0.035), Stroop I, II and III (I: *p *= 0.017; II: *p *< 0.001; III: *p *= 0.021), categorical fluency (*p *= 0.001), WAIS similarities (*p *< 0.001), Happé ToM (*p *= 0.011), and RAVLT immediate (*p *= 0.004) and delayed recall (*p *= 0.030). Converters with a *GRN* mutation deteriorated significantly on attention and mental processing speed, and executive function (Fig. 2b, c; Table 3). Within these domains, performances on TMT-B (*p *< 0.001), Stroop III (*p *< 0.001), WCST (*p *= 0.005), letter fluency (*p *= 0.012) and WAIS similarities (*p *< 0.001) deteriorated significantly over time. Converters with bvFTD had a similar pattern of cognitive decline as *MAPT* converters, with lower scores on social cognition, memory, language, attention and executive function (Table 3). Comparably, converters with nfvPPA had a similar pattern of cognitive decline as *GRN* converters, with lower scores on attention and executive function (Table 3). There were no differences in decline between converters with bvFTD and nfvPPA (Table 3). The raw neuropsychological test scores per time point can be found in Supplementary Table 3.

**Table 3:** Cognitive trajectories in *MAPT*, *GRN*, bvFTD and nfvPPA converters, and non-converters

Domain test	*MAPT* converters (*n* = 5)			*GRN* converters (*n* = 3)			bvFTD converters (*n* = 6)			nfvPPA converters (n = 2)			Non-converters (= 38)		
	Baseline	*β*	*p*	Baseline	*β*	*p*	Baseline	*β*	*p*	Baseline	*β*	*p*	Baseline	*β*	*p*
Language	0.1 ± 0.7	− 0.028	**<** **0.001**^a^	0.6 ± 0.2	− 0.007	0.299	0.1 ± 0.7	− 0.025	**<** **0.001**^a^	0.6 ± 0.2	− 0.014	0.061	0.1 ± 0.6	0.002	0.408
BNT	54.3 ± 6.9	− 0.239	**<** **0.001**^a^	57.5 ± 2.1	− 0.019	0.604	54.3 ± 6.9	− 0.224	**<** **0.001**^a^	57.5 ± 2.1	− 0.033	0.396	54.2 ± 4.2	− 0.001	0.960
SAT	27.0 ± 1.4	− 0.040	**0.034**	28.0 ± 1.4	0.006	0.805	27.0 ± 1.4	− 0.036	0.052	28.0 ± 1.4	0.000	0.993	27.7 ± 2.0	0.013	0.127
ScreeLing phonology	24.0 ± 0.0	0.002	0.617	24.0 ± 0.0	− 0.011	0.114	24.0 ± 0.0	0.004	0.358	24.0 ± 0.0	− 0.017	**0.018**	23.8 ± 0.4	− 0.002	0.551
Categorical fluency	25.8 ± 4.6	− 0.250	**<** **0.001**^a^	28.0 ± 2.8	− 0.149	**0.022**	25.8 ± 4.6	− 0.237	**<** **0.001**^a^	28.0 ± 2.8	− 0.170	**0.015**	24.0 ± 6.3	0.014	0.546
Attention and mental processing speed	0.3 ± 0.6	− 0.010	**0.006**	0.2 ± 0.3	− 0.013	**0.005**	0.3 ± 0.6	− 0.010	**0.004**	0.2 ± 0.3	− 0.013	**0.006**	0.1 ± 0.8	− 0.001	0.321
TMT part A^b^	20.0 ± 6.3	0.067	0.448	25.0 ± 8.5	0.073	0.539	20.0 ± 6.3	0.065	0.449	25.0 ± 8.5	0.090	0.483	31.1 ± 11.8	0.051	0.181
Stroop card I^b^	44.0 ± 5.2	0.101	**0.030**	46.5 ± 6.4	0.058	0.349	44.0 ± 5.2	0.106	**0.020**	46.5 ± 6.4	0.044	0.503	44.4 ± 8.9	− 0.020	0.345
Stroop card II^b^	58.5 ± 7.6	0.331	**<** **0.001**^a^	56.5 ± 0.7	0.186	**0.006**	57.5 ± 7.6	0.319	**<** **0.001**^a^	56.5 ± 0.7	0.194	**0.008**	58.8 ± 12.9	− 0.032	0.217
Digit Span forwards	9.5 ± 1.7	0.010	0.609	9.0 ± 0.0	− 0.038	0.146	9.5 ± 1.7	0.010	0.601	9.0 ± 0.0	− 0.043	0.119	9.3 ± 2.6	− 0.013	0.088
LDST	34.8 ± 6.7	− 0.100	**0.012**	35.0 ± 0.0	− 0.061	0.235	34.8 ± 6.7	− 0.098	**0.011**	35.0 ± 0.0	− 0.061	0.270	33.3 ± 6.9	0.004	0.809
Executive function	0.6 ± 0.4	− 0.018	**<** **0.001**^a^	0.6 ± 0.1	− 0.032	<0.001	0.6 ± 0.4	− 0.020	**<** **0.001**	0.6 ± 0.1	− 0.029	**<** **0.001**^a^	0.2 ± 0.8	− 0.001	0.515
TMT part B^b^	57.0 ± 27.0	0.472	**0.038**	48.0 ± 32.5	1.448	**<0.001** ^a^	57.0 ± 27.0	0.684	**0.006**	48.0 ± 32.5	0.921	**0.010**	71.2 ± 40.4	− 0.132	0.195
Stroop card III^b^	87.5 ± 23.4	0.468	**<** **0.001**^a^	86.5 ± 7.8	0.734	**<0.001** ^a^	87.5 ± 23.4	0.449	**<** **0.001**^a^	86.5 ± 7.8	0.815	**<** **0.001**^a^	93.7 ± 24.8	− 0.026	0.577
Digit span backwards	8.0 ± 1.4	− 0.018	0.284	5.5 ± 0.7	− 0.039	0.082	8.0 ± 1.4	− 0.022	0.186	5.5 ± 0.7	− 0.033	0.172	6.5 ± 2.0	− 0.003	0.721
WCST concepts	6.0 ± 0.0	− 0.015	0.193	6.0 ± 0.0	− 0.040	**0.007**	6.0 ± 0.0	− 0.021	0.073	6.0 ± 0.0	− 0.032	**0.035**	5.7 ± 0.8	− 0.006	0.323
Letter fluency	35.8 ± 7.9	− 0.143	0.101	45.5 ± 17.7	− 0.328	**0.010**	35.8 ± 7.9	− 0.156	0.066	45.5 ± 17.7	− 0.339	**0.013**	37.9 ± 13.0	− 0.048	0.245
Similarities	29.0 ± 1.2	− 0.151	**<** **0.001**^a^	29.0 ± 1.4	− 0.175	**<0.001** ^a^	29.0 ± 1.2	− 0.155	**<** **0.001**^a^	29.0 ± 1.4	− 0.175	**<** **0.001**^a^	25.5 ± 4.0	0.004	0.775
Social cognition	0.0 ± 1.0	− 0.022	**<** **0.001**^a^	0.8 ± 0.1	− 0.012	0.127	0.0 ± 1.0	− 0.021	**<** **0.001**^a^	0.8 ± 0.1	− 0.016	0.071	0.3 ± 0.7	− 0.002	0.336
Happé ToM	12.3 ± 5.1	− 0.096	**0.002** ^a^	13.5 ± 2.1	0.017	0.672	12.3 ± 5.1	− 0.078	**0.012**	13.5 ± 2.1	− 0.019	0.669	12.8 ± 3.0	− 0.012	0.380
Happé non-Tom	12.3 ± 2.4	− 0.067	**0.010**	15.5 ± 0.7	− 0.041	0.215	12.3 ± 2.4	− 0.060	**0.016**	15.5 ± 0.7	− 0.062	0.080	12.8 ± 2.7	− 0.012	0.267
Ekman faces	43.5 ± 6.1	− 0.089	**0.023**	50.0 ± 0.0	− 0.175	**0.001** ^a^	43.5 ± 6.1	− 0.118	**0.003**	50.0 ± 0.0	− 0.127	**0.024**	47.3 ± 5.4	− 0.001	0.965
Memory	− 1.0 ± 2.0	− 0.050	**<** **0.001**^a^	0.7 ± 0.8	0.002	0.751	− 1.0 ± 2.0	− 0.044	**<** **0.001**^a^	0.7 ± 0.8	− 0.005	0.525	0.2 ± 0.8	− 0.002	0.473
VAT	10.0 ± 2.4	− 0.030	**0.005**	12.0 ± 0.0	0.004	0.675	10.0 ± 2.4	− 0.027	**0.011**	12.0 ± 0.0	0.000	0.983	11.6 ± 0.8	− 0.002	0.705
RAVLT imm. recall	42.5 ± 9.1	− 0.241	**0.001** ^a^	54.5 ± 19.1	− 0.111	0.226	42.5 ± 9.1	− 0.210	**0.003**	54.5 ± 19.1	− 0.177	0.067	46.7 ± 10.0	− 0.009	0.797
RAVLT del. recall	7.5 ± 5.5	− 0.085	**<** **0.001**^a^	10.5 ± 5.0	0.002	0.951	7.5 ± 5.5	− 0.080	**<** **0.001**^a^	10.5 ± 5.0	− 0.002	0.954	9.7 ± 3.2	− 0.009	0.359
RAVLT recognition	27.3 ± 3.1	− 0.037	**0.005**	30.0 ± 0.0	− 0.014	0.266	27.3 ± 3.1	− 0.036	**0.004**	30.0 ± 0.0	− 0.014	0.308	29.3 ± 1.1	− 0.009	0.461
Visuoconstruction	0.2 ± 0.8	− 0.009	0.217	0.2 ± 0.2	− 0.010	0.312	0.2 ± 0.8	− 0.008	0.250	0.2 ± 0.2	− 0.013	0.237	− 0.1 ± 1.0	0.000	0.895
Block design	51.0 ± 27.1	− 0.222	0.064	32.0 ± 1.4	− 0.148	0.333	51.0 ± 27.1	− 0.235	**0.042**	32.0 ± 1.4	− 0.109	0.503	37.1 ± 18.5	− 0.006	0.898
Clock drawing	11.8 ± 2.1	− 0.002	0.876	13.5 ± 0.7	− 0.014	0.459	11.8 ± 2.1	− 0.001	0.966	13.5 ± 0.7	− 0.023	0.281	12.3 ± 1.6	0.001	0.888

Values indicate: mean ± standard deviation; *β* represents estimate of change over time. Composite domain scores are *z*-scores, individual test scores are raw scores. Composite domain scores are expressed as z-scores, the individual test scores are raw scores. *p* values represent comparisons to non-converters. Significant comparisons are displayed in bold

*MAPT* microtubule-associated protein tau, *GRN* progranulin, *bvFTD* behavioural variant frontotemporal dementia, *nfvPPA* non-fluent variant primary progressive aphasia, *BNT* Boston Naming Test, *SAT* semantic association test, *TMT* Trail making Test, *WAIS* Wechsler Adult Intelligence Scale, *LDST* letter digit, substitution test, *WCST* Wisconsin card sorting test, *ToM* theory of mind, *VAT* visual association test, *RAVLT* Rey Auditory Verbal Learning Test, *imm* immediate, *del* delayed

^a^Survived Bonferroni correction for multiple comparisons

^b^Higher scores and *β* weights indicate worse performance

### Classification between converters and non-converters

Between 4 and 2 years before symptom onset, the delta domain and individual neuropsychological test scores failed to distinguish significantly between converters and non-converters. Between 2 years before symptom onset and symptom onset decline on categorical fluency was predictive of an underlying *MAPT* mutation (*p *= 0.025; Table 4). Decline on ScreeLing phonology (*p *= 0.046) and letter fluency (*p *= 0.046) was predictive of conversion to nfvPPA (Table 4).

**Table 4:** ROC analyses on neuropsychological decline between 2 years before conversion and symptom onset in converters

Domain and individual neuropsychological tests	bvFTD vs. nfvPPA converters						*MAPT* vs. *GRN* converters					
	AUC	95% CI	*p*	Optimal Δ^a^	Sensitivity (%)	Specificity (%)	AUC	95% CI	*p*	Optimal Δ^b^	Sensitivity (%)	Specificity (%)
Language	0.667	0.29–1.00	0.505	–	–	–	0.867	0.51–1.00	0.101	–	–	–
BNT	0.708	0.34–1.00	0.405	–	–	–	0.90	0.67–1.00	0.074	–	–	–
SAT	0.625	0.24–1.00	0.617	–	–	–	0.833	0.54–1.00	0.136	–	–	–
ScreeLing phonology	1.000	1.00–1.00	**0.046**	− 0.5	100	100	0.700	0.21–1.00	0.371	–	–	–
Categorical fluency	0.833	0.53–1.00	0.182	–	–	–	1.000	1.00–1.00	**0.025**	− 6.5	100	100
Attention and mental processing speed	0.750	0.41–1.00	0.317	–	–	–	0.600	0.19–1.00	0.655	–	–	–
TMT part A	0.542	0.00–1.00	0.868	–	–	–	0.50	0.05–0.95	1.000	–	–	–
Stroop card I	0.583	0.19–0.97	0.739	–	–	–	0.600	0.17–1.00	0.655	–	–	–
Stroop card II	0.583	0.12–1.00	0.739	–	–	–	0.667	0.22–1.00	0.456	–	–	–
Digit Span forwards WAIS-III	0.750	0.40–1.00	0.317	–	–	–	0.633	0.23–1.00	0.551	–	–	–
LDST	0.625	0.23–1.00	0.617	–	–	–	0.633	0.22–1.00	0.551	–	–	–
Executive function	0.583	0.19–0.98	0.739	–	–	–	0.733	0.36–1.00	0.297	–	–	–
TMT part B	0.667	0.29–1.00	0.617	–	–	–	0.900	0.64–1.00	0.121	–	–	–
Stroop card III	0.833	0.51–1.00	0.182	–	–	–	0.600	0.15–1.00	0.655	–	–	–
Digit span backwards WAIS-III	0.542	0.09–1.00	0.868	–	–	–	0.567	0.14–0.99	0.766	–	–	–
WCST concepts	0.500	0.10–0.90	1.000	–	–	–	0.700	0.32–1.00	0.371	–	–	–
Letter fluency	1.000	1.00–1.00	**0.046**	− 16	100	100	0.767	0.36–1.00	0.233	–	–	–
Similarities WAIS-III	0.625	0.14–1.00	0.617	–	–	–	0.567	0.13–1.00	0.766	–	–	–
Social cognition	0.500	0.00–1.00	1.000	–	–	–	0.667	0.13–1.00	0.456	–	–	–
Happé ToM	0.458	0.00–1.00	0.868	–	–	–	0.700	0.21–1.00	0.371	–	–	–
Happé non-Tom	0.500	0.00–1.00	1.000	–	–	–	0.667	0.22–1.00	0.456	–	–	–
Ekman faces	0.667	0.15–1.00	0.505	–	–	–	0.567	0.07–1.00	0.766	–	–	–
Memory	0.750	0.41–1.00	0.317	–	–	–	0.933	0.75–1.00	0.053	–	–	–
VAT	0.792	0.45–1.00	0.243	–	–	–	0.933	0.75–1.00	0.053	–	–	–
RAVLT immediate recall	0.667	0.15–1.00	0.505	–	–	–	0.600	0.09–1.00	0.655	–	–	–
RAVLT delayed recall	0.667	0.27–1.00	0.505	–	–	–	0.867	0.58–1.00	0.101	–	–	–
RAVLT recognition	0.750	0.37–1.00	0.317	–	–	–	0.900	0.65–1.00	0.074	–	–	–
Visuoconstruction	0.583	0.19–0.98	0.739	–	–	–	0.600	0.19–1.00	0.655	–	–	–
Block design WAIS-III	0.808	0.35–1.00	0.405	–	–	–	0.500	0.07–0.93	1.000	–	–	–
Clock drawing	0.667	0.29–1.00	0.505	–	–	–	0.600	0.16–1.00	0.655	–	–	–

*AUC* area under the curve, *CI* confidence interval, *bvFTD* behavioural variant frontotemporal dementia, *nfvPPA* non-fluent variant frontotemporal dementia, *MAPT* microtubule-associated protein tau, *GRN* progranulin, *BNT* Boston Naming Test, *SAT* semantic association test, *TMT* Trail making Test, *WAIS* Wechsler Adult Intelligence Scale, *LDST* letter digit substitution test, *WCST* Wisconsin Card Sorting Test, *ToM* theory of mind, *VAT* visual association test, *RAVLT* Rey Auditory Verbal Learning Test

^a^Negative delta represents decline in test performance in nfvPPA vs. bvFTD (i.e. when a converter declines on this particular task, he/she is more likely to develop nfvPPA

^b^Negative delta represents decline in test performance in *MAPT* vs *GRN* (i.e. when a converter declines on this particular task, he/she is more likely to have a underlying *MAPT* mutation

## Discussion

This study examined a large cohort of at-risk participants from *GRN* and *MAPT* FTD families by means of neuropsychological assessment during a 4-year follow-up. Within the study time window, eight mutation carriers became symptomatic. Converters with a *MAPT* and *GRN* mutation had mutual as well as gene-specific profiles of cognitive decline. Cognitive decline on categorical fluency between 2 years before conversion and symptom onset was predictive for an underlying *MAPT* mutation, and decline on ScreeLing phonology and letter fluency was predictive for conversion to nfvPPA. These results suggest that neuropsychological assessment could provide sensitive clinical biomarkers to identify and track FTD mutation carriers at-risk of converting to the symptomatic stage. These findings hold potential for improving early clinical diagnosis by identifying the most sensitive neuropsychological tests for conversion, and use in upcoming disease-modifying clinical trials.

Following the *MAPT* mutation carriers over a 4-year period, we found significant decline in language, social cognition and memory. This is consistent with findings from previous presymptomatic familial FTD studies, in which both cross-sectional [9–11, 33] and longitudinal [8] decline was found. Specifically, in our first follow-up study [8], we demonstrated decline in the domains language, social cognition and memory 5–8 years before estimated symptom onset. It should be taken into account that this study made use of estimated onset as a proxy, instead of actual symptom onset as in the present study—but the similar profile of decline confirms the presence of early changes in these three domains. As in our previous study, the present results are largely driven by the converters. This could suggest that neuropsychological test scores remain static while mutation carriers are presymptomatic, and cognitive decline starts only near or at symptom onset [34–36], suggesting an explosive rather than gradual start of the symptomatic disease stage. Alternatively, we might be unable to pick up subtle cognitive changes in presymptomatic mutation carriers due to lack of power. Also, although well-validated, most of our neuropsychological tests were not developed for repeated administration in a preclinical population [37]. We therefore cannot rule out that familiarity and/or practice effects are obscuring subtle cognitive decline, a notion that seems to be underwritten by improvement in social cognition and memory in controls, but not mutation carriers.

In our exploratory analyses in converters, we discovered both common as well as mutation-specific profiles of cognitive decline in *MAPT* and *GRN*. In both mutations, decline in attention, mental processing speed and executive function was found—while only converters with a *MAPT* mutation demonstrated decline on language, memory and social cognition. Previous studies in familial FTD also point to distinct profiles for *MAPT* and *GRN* [8, 10–12], and are largely consistent with our present findings. Another important aspect is the longitudinal tracking of the different clinical phenotypes. The similar patterns of cognitive decline in bvFTD as *MAPT*, and nfvPPA as *GRN* are related to the dominant genotype in each group (e.g. all nfvPPA converters have a *GRN* mutation). These findings suggest that neuropsychological assessment can be used to track the different mutations and phenotypes from the presymptomatic to the symptomatic stage, which is advantageous considering the need for good clinical endpoints in future disease-modifying trials.

Extending the findings from our first follow-up study [8], we demonstrated significant decline on the RAVLT recall in presymptomatic *MAPT* mutation carriers. The additional finding that lower memory scores over time were also found in *MAPT*, and not *GRN* converters—suggesting a mutation-specific aetiology—corroborate this. Although memory loss has been described in GRN [38, 39], this is usually a later symptom, while episodic memory impairment has been found as the presenting and most prominent symptom in MAPT [7, 40, 41]. Interestingly, the Genetic Frontotemporal dementia Initiative (GENFI) consortium revealed hippocampal atrophy in presymptomatic *MAPT* from 15 years before estimated symptom onset [10], and as this medial temporal structure is critical for episodic memory processing [42] this offers a good explanation for our findings. In line with earlier studies [42, 43], we did find deficits in verbal recall but not visual associative memory. Semantically loaded tasks such as the RAVLT can be particularly more difficult than visual memory tasks like the VAT, as a result of the prominent semantic impairments seen early in *MAPT*-associated FTD [44]. Our results contribute to the present thinking that memory deficits can be an integral part of the clinical spectrum [42], and comprehensive memory tasks should therefore be incorporated in the standard diagnostic work-up.

Knowing the cognitive profile of decline indicative for conversion is important to get more insight into the timing of clinical changes in the earliest disease stage. We found that conversion can be predicted based on cognitive decline in the 2 years prior to symptom onset, but not earlier. As the cognitive decline was part of the diagnostic process of determining conversion, this is not a surprising finding. However, it does suggest a more explosive disease development with cognitive decline accelerating rapidly in proximity of symptom onset, which is in line with evidence from a large familial Alzheimer’s disease cohort [45]. By selectively choosing tests within the domains that have prognostic abilities, the neuropsychological battery can be shortened, which would benefit patient burden and helps cutting healthcare expenses. Especially fluency tasks seem to be promising candidates, as they were able to distinguish accurately between future phenotype and underlying genotype. The latter is essential for patient stratification in future clinical trials targeting specific pathologies, and ideally these interventions should be applied in the presymptomatic stage [46]. Reliable phenotypic prediction furthermore optimizes the diagnostic process by shortening the current diagnostic delay [47], and is helpful for the patient, caregiver and clinician in knowing what disease presentation and course to expect. Verbal fluency tests are widely used in dementia diagnosis setting [48], and are affected in both presymptomatic [8, 11] and symptomatic FTD [49, 50]. Future research could additionally investigate the use of qualitative assessment of verbal fluency (e.g. clustering, switching between clusters), as recent research [49] points to differences between FTD and PPA subtypes—making this a promising application of verbal fluency for a precise clinical differentiation in presymptomatic and early stage FTD.

Key strengths of our study constitute our longitudinal design, spanning a 4-year follow-up of at-risk participants from both *MAPT* and *GRN* families. Although our group of converters is currently small, this is the first study tracking FTD mutation carriers from the presymptomatic to symptomatic disease stage. Being aware of the caveats of small sample sizes and administering a large amount of neuropsychological tests with respect to statistical power, our results warrant replication in our cohort as well as larger international cohorts such as GENFI [10], in which with the passing of time more mutation carriers will approach symptom onset and/or convert to clinical FTD. The dropout rate is very low, creating balanced datasets across the three time points. Additionally, use of multilevel linear modeling further handles efficiently with missing data. Directions for future research entail the development of neuropsychological tasks more suited to administer in the presymptomatic phase (robust to ceiling effects) and repeated administration (robust to practice and able to measure small changes). More extensive quantification tools of behavioural functioning are also needed to capture the entire clinical spectrum of (presymptomatic) FTD, as well as assessment methods that rely less on the accuracy of informant report [37]. A disadvantage of the study is the fact that the neuropsychological assessment was part of the clinical assessment with which we determined conversion to the symptomatic stage. This has likely led to a circular reasoning, as we demonstrated that converters declined over time, while cognitive decline was considered a prerequisite for conversion. Ideally, the tests assessed in our study should not have been used in the diagnosis of conversion. However, in our multidisciplinary meeting, we followed the international consensus criteria for bvFTD [3] and PPA [6], using all available clinical information—e.g. MR imaging of the brain, anamnestic and heteroanamnestic information, behavioural and neuropsychiatric questionnaires, unblinding of genetic status—so that symptom onset did not solely depend on the neuropsychological assessment. Furthermore, as the multilevel model assumes a linear relationship between genetic status and cognitive decline over time, we could have missed non-linear effects over time. Lastly, the analyses on the non-converters and controls were performed using the original baseline and follow-up visits, regardless of, e.g. age and time to estimated symptom onset. It is possible that these analyses therefore lost some sensitivity to detect cognitive decline over time. However, as between-group analyses on age and estimated years to symptom onset in converters, non-converters, and controls did not show significant differences (respectively, *p *= 0.99 and *p *= 0.19), we believe this effect is minimal.

Our study investigates longitudinal neuropsychological performance in a large cohort of at-risk individuals from genetic FTD families. We provide evidence of mutation-specific cognitive decline when moving from the presymptomatic into symptomatic stage, and of neuropsychological trajectories predicting symptom onset. These results suggest the potential biomarker value of neuropsychological assessment in both disease-monitoring and predicting conversion to clinical FTD.

## Electronic supplementary material

Below is the link to the electronic supplementary material.

Supplementary material 1 (DOCX 66 kb)

Supplementary material 2 (DOCX 16 kb)
