# Supplementary material for: Longitudinal cognitive biomarkers predicting symptom onset in presymptomatic frontotemporal dementia
Source: J Neurol. 2018 Apr 7;265(6):1381–92. doi: 10.1007/s00415-018-8850-7 (PMC5990575; doi:10.1007/s00415-018-8850-7)
Supplement: Supplementary file 1 — Supplementary material 1 (DOCX 66 kb) [file 415_2018_8850_MOESM1_ESM.docx]

| **Demographics**  **Supplementary Table 1. Demographic, clinical and neuropsychological data of the converters at symptom onset.** | **Converter** | | | | | | | |
| --- | --- | --- | --- | --- | --- | --- | --- | --- |
|  | **1** | **2** | **3** | **4** | **5** | **6** | **7** | **8** |
| Clinical diagnosis | bvFTD | bvFTD | bvFTD | nfvPPA | nfvPPA | bvFTD | bvFTD | bvFTD |
| Age at onset | 67 | 56 | 57 | 51 | 57 | 42 | 45 | 43 |
| Mean onset age family | 59.7 | 53.2 | 53.2 | 59.7 | 59.7 | 44.5 | 44.5 | 44.5 |
| Gene | *GRN* | *MAPT* | *MAPT* | *GRN* | *GRN* | *MAPT* | *MAPT* | *MAPT* |
| Mutation | Ser82fs | P301L | P301L | Ser82fs | Ser82fs | G272V | G272V | G272V |
| Gender | Female | Female | Male | Female | Female | Male | Male | Male |
| Study visit on which diagnosis was set | Follow-up 1 | Follow-up 1 | Follow-up 2 | Follow-up 2 | Follow-up 2 | Follow-up 2 | Follow-up 2 | Follow-up 2 |
| **Global cognition and questionnaires** | | | | | | | | |
| MMSE (/30) | 28 | 30 | 27 | 29 | 26 | 26 | 26 | 27 |
| FAB (/18) | 16 | 16 | 13 | 18 | 15 | 15 | 16 | 17 |
| NPI (/144) | 29 | 0 | 23 | 2 | 1 | 15 | 1 | 39 |
| CBI-R (/180) | 46 | 0 | 32 | 4 | 0 | 42 | 25 | 55 |
| **Neuropsychological test** | | | | | | | | |
| ***Social cognition*** | | | | | | | | |
| Happé Cartoon Test ToM | -1.3 | -0.7 | -2.2 | 1.0 | -1.0 | -2.2 | -0.7 | -0.4 |
| Happé Cartoon Test non-ToM | -2.1 | -2.1 | -1.8 | 1.2 | -1.4 | -2.5 | -1.4 | 0.8 |
| Ekman Faces Test | -0.3 | -2.4 | -1.1 | 1.0 | -1.9 | -2.1 | -0.6 | 0.3 |
| ***Language*** | | | | | | | | |
| Boston Naming Test 60-item | -0.4 | 0.0 | -0.4 | 0.7 | 0.3 | -7.1 | -3.1 | 0.5 |
| ScreeLing phonology | -0.1 | 0.5 | 0.5 | -0.6 | -0.6 | 0.5 | 0.5 | 0.5 |
| SAT | 0.9 | 0.9 | -1.0 | 0.3 | 0.9 | -4.1 | 0.3 | 0.3 |
| Categorical fluency (animals) | -1.3 | -1.3 | -1.8 | -0.1 | -1.3 | -2.0 | -1.2 | -1.3 |
| ***Executive function*** | | | | | | | | |
| Letter fluency | -1.7 | -2.4 | -1.2 | -0.7 | -0.9 | -0.8 | 0.0 | -0.3 |
| TMT part B | -3.6 | -2.7 | -1.1 | -0.5 | >-3.0 | 0.3 | 0.9 | 0.1 |
| Stroop card III | -2.5 | -1.7 | -3.1 | -2.4 | -1.7 | 0.3 | 0.4 | -1.1 |
| WCST | -3.2 | -3.2 | -2.3 | -1.4 | -1.4 | 0.3 | 0.3 | 0.3 |
| Similarities WAIS-III | -1.0 | -1.2 | -1.2 | 0.1 | -1.8 | -1.0 | 0.9 | -0.7 |
| Digit Span backwards WAIS-III | -1.3 | -1.3 | -0.3 | -0.8 | -1.8 | 1.3 | 1.8 | -0.8 |
| ***Attention & mental processing speed*** | | | | | | | | |
| TMT part A | -1.8 | -1.7 | -1.5 | 0.6 | -1.2 | 1.3 | 1.1 | 0.3 |
| Stroop card I | -1.1 | 0.6 | -0.6 | -0.2 | -0.2 | 0.0 | 0.6 | -0.9 |
| Stroop card II | -0.8 | -0.9 | -3.6 | -0.8 | -0.6 | -0.7 | -0.3 | -1.2 |
| LDST | -0.9 | -0.6 | -1.4 | 0.0 | -0.9 | 0.0 | 0.7 | -2.0 |
| Digit Span forwards WAIS-III | -0.6 | -0.6 | -1.3 | -1.3 | -2.6 | 0.1 | 2.2 | 0.1 |
| ***Memory*** | | | | | | | | |
| RAVLT immediate recall | 0.2 | 0.0 | -1.9 | 1.7 | -1.4 | -1.7 | -0.1 | -1.5 |
| RAVLT delayed recall | -0.1 | -0.4 | -2.1 | 1.3 | -0.4 | -3.8 | -0.4 | -1.8 |
| RAVLT recognition | 0.6 | -0.2 | -0.2 | 0.6 | -0.2 | -8.2 | -0.2 | -1.8 |
| Visual Association Test | 0.4 | 0.4 | -2.3 | 0.4 | 0.4 | -2.9 | -2.3 | 0.4 |
| ***Visuoconstruction*** | | | | | | | | |
| Clock drawing | 0.2 | 0.2 | 0.2 | 0.2 | -0.9 | -1.9 | -0.9 | 0.2 |
| Block Design WAIS-III | -2.0 | -1.6 | -0.4 | -0.2 | -1.6 | -0.4 | 1.7 | -0.4 |

Values indicate z-scores (i.e. individual test score minus the mean of healthy controls, divided by the standard deviation of healthy controls). Abbreviations: bvFTD, behavioural variant frontotemporal dementia; nfvPPA, nonfluent variant primary progressive aphasia; *GRN*, progranulin; *MAPT*, microtubule-associated protein tau; MMSE, Mini-Mental State Examination; FAB, frontal assessment battery; NPI-Q, Neuropsychiatric Inventory; CBI-R, Cambridge Behavioural Inventory-Revised; ToM, theory of mind; SAT, Semantic Association Test; TMT, Trailmaking Test; WCST, Wisconsin Card Sorting Test; WAIS, Wechsler Adult Intelligence Scale; LDST, Letter Digit Substitution Test; RAVLT, Rey Auditory Verbal Learning Test. Cognitive disorders (≥2 SD below mean) are highlighted in red; values below average (-2 ≤ SD ≥ -1) are highlighted in orange. Follow-up 1 = visit 2 years after study entry; follow-up 2 = visit 4 years after study entry.

**Supplementary Table 2. Raw neuropsychological test scores of healthy controls, *MAPT* mutation carriers, and *GRN* mutation carriers at four years before symptom onset (-4), two years before symptom onset (-2) and at symptom onset (0).**

| **Cognitive domains  and individual tests**  **Supplementary Table 2. Raw neuropsychological test scores of healthy controls, *MAPT* mutation carriers, and *GRN* mutation carriers at four years before symptom onset (-4), two years before symptom onset (-2) and at symptom onset (0).** | **healthy controls (n=39)** | | | ***MAPT* mutation carriers (n=15)** | | | ***GRN* mutation carriers (n=31)** | | |
| --- | --- | --- | --- | --- | --- | --- | --- | --- | --- |
|  | **-4** | **-2** | **0** | **-4** | **-2** | **0** | **-4** | **-2** | **0** |
| **Language** | 0.0 ± 0.6 | 0.0 ± 0.5 | 0.0 ± 0.7 | 0.2 ± 0.6 | 0.1 ± 0.6 | -0.3 ± 1.0 | 0.1 ± 0.7 | 0.0 ± 0.4 | 0.3 ± 0.4 |
| **BNT** | 53.4 ± 4.5 | 54.4 ± 8.0 | 54.8 ± 4.5 | 52.6 ± 5.3 | 53.0 ± 7.1 | 50.9 ± 9.4 | 55.1 ± 3.7 | 56.6 ± 2.6 | 56.6 ± 2.2 |
| **SAT** | 27.8 ± 1.1 | 28.4 ± 1.3 | 27.6 ± 1.6 | 27.9 ± 1.5 | 28.5 ± 2.0 | 27.5 ± 2.2 | 27.5 ± 2.0 | 27.9 ± 1.4 | 28.5 ± 1.0 |
| **ScreeLing phonology** | 23.5 ± 0.8 | 23.7 ± 0.7 | 23.6 ± 0.9 | 23.9 ± 0.3 | 24.0 ± 0.1 | 23.7 ± 0.6 | 23.8 ± 0.5 | 23.8 ± 0.4 | 23.7 ± 0.5 |
| **Categorical fluency** | 23.9 ± 4.9 | 24.5 ± 6.4 | 25.4 ± 6.4 | 26.5 ± 6.6 | 25.6 ± 4.9 | 23.3 ± 7.5 | 23.4 ± 5.7 | 24.2 ± 5.3 | 25.3 ± 5.9 |
| **Attention & processing speed** | 0.0 ± 0.8 | 0.0 ± 0.8 | 0.0 ± 0.8 | 0.3 ± 0.6 | 0.3 ± 0.5 | 0.0 ± 0.9 | 0.1 ± 0.9 | 0.0 ± 0.9 | -0.3 ± 0.9 |
| **TMT part A*** | 31.8 ± 15.0 | 30.5 ± 12.9 | 28.7 ± 10.4 | 26.1 ± 9.7 | 25.6 ± 7.3 | 29.1 ± 11.8 | 31.4 ± 12.2 | 31.6 ± 12.0 | 35.5 ± 13.4 |
| **Stroop card I*** | 47.1 ± 8.0 | 47.6 ± 8.3 | 48.4 ± 10.1 | 43.2 ± 8.8 | 42.3 ± 6.4 | 44.2 ± 7.8 | 45.0 ± 8.4 | 45.9 ± 8.1 | 47.9 ± 8.4 |
| **Stroop card II*** | 58.5 ± 10.6 | 58.7 ± 10.9 | 56.0 ± 11.1 | 54.9 ± 8.5 | 53.5 ± 7.0 | 57.7 ± 15.6 | 60.2 ± 13.2 | 60.0 ± 12.9 | 61.3 ± 13.5 |
| **Digit Span forwards** | 8.7 ± 1.9 | 9.0 ± 1.8 | 8.8 ± 1.4 | 9.0 ± 2.6 | 9.5 ± 2.2 | 8.6 ± 2.5 | 9.4 ± 2.4 | 9.0 ± 2.4 | 8.4 ± 1.7 |
| **LDST** | 34.5 ± 6.8 | 34.8 ± 8.1 | 35.2 ± 6.7 | 34.2 ± 4.7 | 33.1 ± 6.3 | 33.6 ± 7.7 | 33.2 ± 7.4 | 33.1 ± 6.8 | 33.4 ± 6.6 |
| **Executive function** | 0.0 ± 0.7 | 0.0 ± 0.6 | 0.0 ± 0.6 | 0.3 ± 0.6 | 0.2 ± 0.6 | -0.1 ± 0.9 | 0.2 ± 0.8 | 0.2 ± 0.7 | -0.2 ± 0.8 |
| **TMT part B*** | 67.8 ± 29.3 | 69.6 ± 34.4 | 67.6 ± 37.7 | 61.0 ± 28.5 | 61.6 ± 27.7 | 71.9 ± 36.0 | 72.2 ± 42.7 | 65.5 ± 26.4 | 76.6 ± 45.7 |
| **Stroop card III*** | 93.7 ± 22.6 | 92.3 ± 24.1 | 86.4 ± 18.6 | 83.8 ± 14.7 | 83.4 ± 22.3 | 85.6 ± 25.3 | 96.6 ± 26.2 | 92.9 ± 24.5 | 95.1 ± 23.7 |
| **Digit Span backwards** | 6.1 ± 2.0 | 6.5 ± 2.0 | 6.5 ± 2.0 | 6.6 ± 1.8 | 7.4 ± 2.6 | 7.3 ± 2.5 | 6.6 ± 2.1 | 7.2 ± 2.4 | 6.0 ± 1.8 |
| **WCST concepts** | 5.5 ± 0.9 | 5.4 ± 1.5 | 5.6 ± 1.1 | 5.6 ± 1.1 | 5.4 ± 1.6 | 5.2 ± 1.5 | 5.8 ± 0.6 | 5.6 ± 0.9 | 5.3 ± 1.1 |
| **Letter fluency** | 32.1 ± 9.9 | 39.5 ± 13.9 | 40.4 ± 11.3 | 36.1 ± 14.3 | 39.0 ± 14.0 | 35.1 ± 14.3 | 38.9 ± 12.0 | 44.1 ± 15.4 | 40.8 ± 14.2 |
| **Similarities** | 24.8 ± 4.7 | 25.2 ± 5.2 | 25.4 ± 4.6 | 25.5 ± 4.7 | 24.7 ± 5.3 | 23.4 ± 5.0 | 26.2 ± 5.0 | 27.3 ± 3.8 | 25.6 ± 5.2 |
| **Social cognition** | 0.0 ± 0.8 | 0.0 ± 0.8 | 0.0 ± 0.8 | 0.2 ± 0.7 | 0.0 ± 0.8 | -0.3 ± 1.1 | 0.3 ± 0.7 | 0.2 ± 0.8 | 0.1 ± 0.8 |
| **Happé ToM** | 11.8 ± 3.4 | 12.6 ± 3.2 | 12.5 ± 3.4 | 12.6 ± 3.7 | 13.2 ± 3.0 | 11.0 ± 3.8 | 12.9 ± 2.9 | 13.3 ± 3.8 | 13.2 ± 3.2 |
| **Happé non-Tom** | 11.7 ± 2.9 | 12.7 ± 2.7 | 12.8 ± 2.7 | 12.4 ± 2.8 | 11.6 ± 3.3 | 11.5 ± 3.5 | 13.0 ± 2.6 | 13.1 ± 2.8 | 13.2 ± 2.6 |
| **Ekman Faces** | 45.7 ± 6.4 | 46.7 ± 6.2 | 48.0 ± 6.2 | 47.0 ± 5.5 | 47.8 ± 6.5 | 47.5 ± 8.4 | 47.1 ± 5.5 | 49.0 ± 5.2 | 48.7 ± 5.6 |
| **Memory** | 0.0 ± 0.7 | 0.0 ± 0.7 | 0.0 ± 0.8 | 0.1 ± 1.3 | -0.3 ± 1.5 | -0.9 ± 3.0 | 0.1 ± 0.9 | -0.2 ± 0.9 | 0.0 ± 0.9 |
| **VAT** | 11.8 ± 0.6 | 11.9 ± 0.3 | 11.8 ± 0.4 | 11.4 ± 1.6 | 11.5 ± 1.3 | 10.9 ± 2.9 | 11.5 ± 0.9 | 11.7 ± 0.8 | 11.6 ± 0.8 |
| **RAVLT imm. recall** | 42.6 ± 9.8 | 50.5 ± 8.8 | 51.1 ± 10.7 | 47.5 ± 9.7 | 51.7 ± 10.5 | 51.4 ± 12.2 | 46.3 ± 10.6 | 51.0 ± 9.7 | 53.8 ± 10.2 |
| **RAVLT del. recall** | 8.4 ± 3.2 | 10.5 ± 2.6 | 11.2 ± 3.0 | 9.7 ± 3.9 | 10.7 ± 3.2 | 9.7 ± 4.5 | 9.4 ± 3.3 | 10.9 ± 3.0 | 12.1 ± 2.9 |
| **RAVLT recognition** | 28.6 ± 2.1 | 29.8 ± 4.5 | 29.3 ± 1.3 | 29.0 ± 2.0 | 29.3 ± 1.3 | 28.6 ± 2.9 | 29.2 ± 1.2 | 29.3 ± 1.1 | 29.5 ± 1.1 |
| **Visuoconstruction** | 0.0 ± 0.8 | 0.0 ± 0.8 | 0.0 ± 0.8 | -0.2 ± 0.7 | -0.1 ± 0.7 | -0.4 ± 0.9 | 0.0 ± 1.0 | 0.0 ± 0.8 | -0.1 ± 1.0 |
| **Block Design** | 36.5 ± 14.0 | 38.8 ± 13.6 | 38.9 ± 13.4 | 35.5 ± 20.8 | 36.4 ± 14.6 | 38.3 ± 14.2 | 39.3 ± 18.5 | 38.6 ± 14.2 | 36.0 ± 15.5 |
| **Clock drawing** | 12.6 ± 1.4 | 13.0 ± 1.1 | 12.8 ± 0.9 | 12.2 ± 1.3 | 13.0 ± 0.8 | 12.1 ± 1.1 | 12.4 ± 1.8 | 12.9 ± 0.9 | 12.7 ± 1.0 |

Values indicate: mean ± standard deviation. Composite domain scores are z-scores, individual test scores are raw scores. Abbreviations: *MAPT*, microtubule-associated protein tau; *GRN*, *progranulin*; BNT, Boston Naming Test; SAT, semantic association test; TMT, Trailmaking test; WAIS, Wechsler Adult Intelligence Scale; LDST, letter digit substitution test; WCST, Wisconsin Card Sorting Test; ToM, theory of mind; VAT, visual association test; RAVLT, Rey Auditory Verbal Learning Test, imm, immediate; del, delayed. Composite domain scores are expressed as z-scores, the individual test scores are raw scores. *Higher scores indicate worse performance.

**Supplementary Table 3. Raw neuropsychological test scores of *MAPT* converters, *GRN* converters, bvFTD converters, nfvPPA converters and non-converters at four years before symptom onset.**

| **Cognitive domains  and individual tests** | ***MAPT/bvFTD* converters (n=5)** | | | ***GRN* converters (n=3)** | | | **nfvPPA converters (n=2)** | | | **non-converters (=38)** | | |
| --- | --- | --- | --- | --- | --- | --- | --- | --- | --- | --- | --- | --- |
|  | **-4** | **-2** | **0** | **-4** | **-2** | **0** | **-4** | **-2** | **0** | **-4** | **-2** | **0** |
| **Language** | 0.1 ± 0.7 | 0.0 ± 0.9 | -0.9 ± 1.3 | 0.6 ± 0.2 | 0.0 ± 0.7 | -0.1 ± 0.2 | 0.6 ± 0.2 | 0.1 ± 0.4 | -0.1 ± 0.2 | 0.1 ± 0.6 | 0.1 ± 0.4 | 0.3 ± 0.4 |
| **BNT** | 54.3 ± 6.9 | 53.8 ± 9.4 | 45.8 ± 14.2 | 57.5 ± 2.1 | 56.3 ± 3.8 | 55.7 ± 2.5 | 57.5 ± 2.1 | 55.6 ± 4.1 | 57.0 ± 1.4 | 54.2 ± 4.2 | 55.6 ± 4.1 | 55.9 ± 3.1 |
| **SAT** | 27.0 ± 1.4 | 28.0 ± 2.9 | 26.4 ± 3.2 | 28.0 ± 1.4 | 28.3 ± 1.5 | 28.7 ± 0.6 | 28.0 ± 1.4 | 28.1 ± 1.4 | 28.5 ± 0.7 | 27.7 ± 2.0 | 28.1 ± 1.4 | 28.4 ± 1.1 |
| **ScreeLing phonology** | 24.0 ± 0.0 | 23.9 ± 0.2 | 24.0 ± 0.0 | 24.0 ± 0.0 | 23.7 ± 0.6 | 23.2 ± 0.3 | 24.0 ± 0.0 | 23.9 ± 0.3 | 23.0 ± 0.0 | 23.8 ± 0.4 | 23.9 ± 0.3 | 23.7 ± 0.5 |
| **Categorical fluency** | 25.8 ± 4.6 | 37.4 ± 3.3 | 15.8 ± 2.2 | 28.0 ± 2.8 | 23.0 ± 7.0 | 19.7 ± 4.6 | 28.0 ± 2.8 | 24.8 ± 5.3 | 21.0 ± 5.7 | 24.0 ± 6.3 | 24.8 ± 5.3 | 26.4 ± 5.7 |
| **Attention & mental speed** | 0.3 ± 0.6 | 0.1 ± 0.6 | -0.4 ± 1.0 | 0.2 ± 0.3 | -0.2 ± 0.5 | -0.8 ± 0.4 | 0.2 ± 0.3 | 0.1 ± 0.8 | -0.7 ± 0.6 | 0.1 ± 0.8 | 0.1 ± 0.8 | -0.1 ± 0.9 |
| **TMT part A*** | 20.0 ± 6.3 | 26.6 ± 8.9 | 29.6 ± 14.7 | 25.0 ± 8.5 | 33.7 ± 9.6 | 36.7 ± 13.1 | 25.0 ± 8.5 | 29.8 ± 11.5 | 31.5 ± 13.4 | 31.1 ± 11.8 | 29.8 ± 11.5 | 33.6 ± 13.1 |
| **Stroop card I*** | 44.0 ± 5.2 | 43.4 ± 6.2 | 48.6 ± 6.8 | 46.5 ± 6.4 | 47.3 ± 5.1 | 53.0 ± 5.2 | 46.5 ± 6.4 | 44.7 ± 8.2 | 50.0 ± 0.0 | 44.4 ± 8.9 | 44.7 ± 8.2 | 45.8 ± 8.6 |
| **Stroop card II*** | 58.5 ± 7.6 | 58.0 ± 3.8 | 72.8 ± 14.6 | 56.5 ± 0.7 | 58.3 ± 6.0 | 66.3 ± 1.2 | 56.5 ± 0.7 | 57.9 ± 12.8 | 66.0 ± 1.4 | 58.8 ± 12.9 | 57.9 ± 12.8 | 57.6 ± 13.7 |
| **Digit Span forwards** | 9.5 ± 1.7 | 9.2 ± 2.3 | 9.0 ± 1.9 | 9.0 ± 0.0 | 7.7 ± 0.6 | 6.7 ± 1.5 | 9.0 ± 0.0 | 9.3 ± 2.5 | 6.0 ± 1.4 | 9.3 ± 2.6 | 9.3 ± 2.5 | 8.5 ± 2.0 |
| **LDST** | 34.8 ± 6.7 | 32.6 ± 6.9 | 30.8 ± 7.1 | 35.0 ± 0.0 | 34.0 ± 6.1 | 31.0 ± 3.5 | 35.0 ± 0.0 | 33.1 ± 6.8 | 32.0 ± 4.2 | 33.3 ± 6.9 | 33.1 ± 6.8 | 34.1 ± 7.1 |
| **Executive function** | 0.6 ± 0.4 | 0.3 ± 0.4 | 0.6 ± 1.1 | 0.6 ± 0.1 | 0.0 ± 0.5 | -1.5 ± 0.7 | 0.6 ± 0.1 | 0.2 ± 0.7 | -1.1 ± 0.2 | 0.2 ± 0.8 | 0.2 ± 0.7 | 0.0 ± 0.7 |
| **TMT part B*** | 57.0 ± 27.0 | 56.2 ± 20.9 | 86.6 ± 53.0 | 48.0 ± 32.5 | 64.0 ± 21.1 | 146.0 ± 82.0 | 48.0 ± 32.5 | 65.4 ± 28.0 | 88.0** | 71.2 ± 40.4 | 65.4 ± 28.0 | 68.9 ± 35.0 |
| **Stroop card III*** | 87.5 ± 23.4 | 87.2 ± 15.7 | 105.4 ± 27.2 | 86.5 ± 7.8 | 101.0 ± 24.6 | 127.0 ± 7.8 | 86.5 ± 7.8 | 89.3 ± 25.1 | 124.5 ± 9.2 | 93.7 ± 24.8 | 89.3 ± 25.1 | 86.6 ± 21.7 |
| **Digit Span backwards** | 8.0 ± 1.4 | 7.2 ± 1.6 | 6.8 ± 2.6 | 5.5 ± 0.7 | 5.0 ± 1.0 | 4.0 ± 1.0 | 5.5 ± 0.7 | 7.4 ± 2.6 | 4.0 ± 1.4 | 6.5 ± 2.0 | 7.4 ± 2.6 | 6.6 ± 2.0 |
| **WCST concepts** | 6.0 ± 0.0 | 6.0 ± 0.0 | 4.6 ± 1.9 | 6.0 ± 0.0 | 6.0 ± 0.0 | 3.3 ± 1.2 | 6.0 ± 0.0 | 5.4 ± 1.3 | 4.0 ± 0.0 | 5.7 ± 0.8 | 5.4 ± 1.3 | 5.5 ± 0.9 |
| **Letter fluency** | 35.8 ± 7.9 | 37.4 ± 9.8 | 29.6 ± 10.6 | 45.5 ± 17.7 | 47.0 ± 17.5 | 28.0 ± 6.2 | 45.5 ± 17.7 | 42.8 ± 15.6 | 31.5 ± 2.1 | 37.9 ± 13.0 | 42.8 ± 15.6 | 41.2 ± 14.5 |
| **Similarities** | 29.0 ± 1.2 | 26.6 ± 3.0 | 21.4 ± 5.5 | 29.0 ± 1.4 | 26.3 ± 3.2 | 19.7 ± 6.0 | 29.0 ± 1.4 | 26.5 ± 4.7 | 20.0 ± 8.5 | 25.5 ± 4.0 | 26.5 ± 4.7 | 49.7 ± 4.7 |
| **Social cognition** | 0.0 ± 1.0 | -0.1 ± 0.7 | -1.3 ± 1.0 | 0.8 ± 0.1 | 0.1 ± 1.0 | -0.5 ± 1.4 | 0.8 ± 0.1 | 0.2 ± 0.8 | -0.2 ± 1.8 | 0.3 ± 0.7 | 0.2 ± 0.8 | 0.2 ± 0.7 |
| **Happé ToM** | 12.3 ± 5.1 | 12.6 ± 2.8 | 8.2 ± 3.0 | 13.5 ± 2.1 | 11.3 ± 6.4 | 11.0 ± 4.4 | 13.5 ± 2.1 | 13.5 ± 3.4 | 12.5 ± 5.0 | 12.8 ± 3.0 | 13.5 ± 3.4 | 13.2 ± 3.1 |
| **Happé non-Tom** | 12.3 ± 2.4 | 13.0 ± 2.0 | 9.0 ± 3.5 | 15.5 ± 0.7 | 12.7 ± 4.9 | 10.7 ± 4.7 | 15.5 ± 0.7 | 12.6 ± 3.0 | 12.5 ± 5.0 | 12.8 ± 2.7 | 12.6 ± 3.0 | 13.3 ± 2.4 |
| **Ekman Faces** | 43.5 ± 6.1 | 43.4 ± 5.1 | 40.6 ± 6.9 | 50.0 ± 0.0 | 51.7 ± 5.7 | 45.3 ± 9.0 | 50.0 ± 0.0 | 49.1 ± 5.3 | 45.0 ± 12.7 | 47.3 ± 5.4 | 49.1 ± 5.3 | 49.7 ± 5.6 |
| **Memory** | -1.0 ± 2.0 | -0.9 ± 2.1 | -2.9 ± 1.0 | 0.7 ± 0.8 | -0.2 ± 1.0 | 0.3 ± 0.7 | 0.7 ± 0.8 | -0.1 ± 0.9 | 0.3 ± 1.0 | 0.2 ± 0.8 | -0.1 ± 0.9 | 0.1 ± 0.9 |
| **VAT** | 10.0 ± 2.4 | 11.0 ± 2.2 | 9.4 ± 4.7 | 12.0 ± 0.0 | 11.3 ± 0.6 | 12.0 ± 0.0 | 12.0 ± 0.0 | 11.7 ± 0.7 | 12.0 ± 0.0 | 11.6 ± 0.8 | 11.7 ± 0.7 | 11.6 ± 0.8 |
| **RAVLT imm. recall** | 42.5 ± 9.1 | 48.0 ± 10.2 | 40.0 ± 9.7 | 54.5 ± 19.1 | 54.0 ± 13.5 | 52.7 ± 16.5 | 54.5 ± 19.1 | 51.4 ± 9.7 | 52.5 ± 23.3 | 46.7 ± 10.0 | 51.4 ± 9.7 | 54.9 ± 9.4 |
| **RAVLT del. recall** | 7.5 ± 5.5 | 9.4 ± 3.5 | 6.2 ± 4.1 | 10.5 ± 5.0 | 12.0 ± 2.6 | 12.0 ± 2.6 | 10.5 ± 5.0 | 11.0 ± 3.0 | 12.5 ± 3.5 | 9.7 ± 3.2 | 11.0 ± 3.0 | 12.0 ± 3.1 |
| **RAVLT recognition** | 27.3 ± 3.1 | 29.2 ± 1.8 | 26.6 ± 4.3 | 30.0 ± 0.0 | 30.0 ± 0.0 | 29.7 ± 0.6 | 30.0 ± 0.0 | 29.3 ± 1.1 | 29.5 ± 0.7 | 29.3 ± 1.1 | 29.3 ± 1.1 | 29.6 ± 1.0 |
| **Visuoconstruction** | 0.2 ± 0.8 | 0.3 ± 0.5 | -0.3 ± 0.6 | 0.2 ± 0.2 | -0.3 ± 0.7 | -0.7 ± 0.6 | 0.2 ± 0.2 | -0.1 ± 0.8 | -0.6 ± 0.9 | -0.1 ± 1.0 | -0.1 ± 0.8 | -0.2 ± 1.0 |
| **Block Design** | 51.0 ± 27.1 | 44.0 ± 15.2 | 36.4 ± 15.9 | 32.0 ± 1.4 | 29.3 ± 8.5 | 22.3 ± 13.1 | 32.0 ± 1.4 | 37.8 ± 14.4 | 27.5 ± 13.4 | 37.1 ± 18.5 | 37.8 ± 14.4 | 38.3 ± 14.6 |
| **Clock drawing** | 11.8 ± 2.1 | 13.2 ± 0.4 | 12.4 ± 0.9 | 13.5 ± 0.7 | 13.0 ± 1.0 | 12.7 ± 0.6 | 13.5 ± 0.7 | 12.9 ± 0.9 | 12.5 ± 0.7 | 12.3 ± 1.6 | 12.9 ± 0.9 | 12.6 ± 1.1 |

Values indicate: mean ± standard deviation. Composite domain scores are z-scores, individual test scores are raw scores. Abbreviations: *MAPT*, microtubule-associated protein tau; *GRN*, *progranulin*; BNT, Boston Naming Test; SAT, semantic association test; TMT, Trailmaking test; WAIS, Wechsler Adult Intelligence Scale; LDST, letter digit substitution test; WCST, Wisconsin Card Sorting Test; ToM, theory of mind; VAT, visual association test; RAVLT, Rey Auditory Verbal Learning Test, imm, immediate; del, delayed. Composite domain scores are expressed as z-scores, the individual test scores are raw scores. *Higher scores indicate worse performance. NB: as all *MAPT* converters had a bvFTD phenotype, *MAPT* converters and bvFTD converters are summarized in one column. ** n=1, therefore no mean and SD available.
