# Supplementary material for: Longitudinal cognitive biomarkers predicting symptom onset in presymptomatic frontotemporal dementia
Source: J Neurol. 2018 Apr 7;265(6):1381–92. doi: 10.1007/s00415-018-8850-7 (PMC5990575; doi:10.1007/s00415-018-8850-7)
Supplement: Supplementary file 2 — Supplementary material 2 (DOCX 16 kb) [file 415_2018_8850_MOESM2_ESM.docx]

**E-references**

E1. Folstein MF, Folstein SE and McHugh PR. "Mini-mental state". A practical method for grading the cognitive state of patients for the clinician. J Psychiatr Res, 1975; 12(3): 189-98.

E2. Dubois B, Slachevsky A, Litvan I, Pillon B. The FAB: a Frontal Assessment Battery at bedside. Neurology 2000; 55(11): 1621-6.

E3. Kaplan E, Goodglass H and Weintraub S. The Boston Naming Test. Philadelphia: Lea & Febiger; 1978.

E4. Visch-Brink E, Stronks D and Denes G. Semantische Associatie Test. Lisse: Swets & Zeitlinger; 2005.

E5. Doesborgh SJ, van de Sandt-Koenderman WM, Dippel DW, et al. Linguistic deficits in the acute phase of stroke. J Neurol, 2003; 250(8): 977-82.

E6. Thurstone LLT and Thurstone TG. Primary mental abilities. Chicago: Science Research Associates; 1962.

E7. Army Individual Test Battery, Manual of directions and scoring. Washington, DC: War Department, Adjutant General's office; 1994.

E8. Stroop JR. Studies of interference in serial verbal reactions. Journal of Experimental Psychology 1935; 18: 643-62.

E9. Wechsler D. WAIS-III Nederlandse Bewerking, Technische handleiding. Lisse: Harcourt Test Publishers; 2005.

E10. Jolles J, Houx PJ, Boxtel MPJ van, Ponds RWHM. Maastricht Aging Study: determinants of cognitive aging. Maastricht, the Netherlands: Neuropsych publishers; 1995.

E11. Nelson HE. A modified card sorting test sensitive to frontal lobe defects. Cortex 1976; 12: 313-24.

E12. Happe F, Brownell H and Winner E. Acquired 'theory of mind' impairments following stroke. Cognition, 1999; 70(3): 211-40.

E13. Ekman P and Friesen WV. Pictures of facial affect. Palo Alto, CA: Consulting psychologists press; 1976.

E14. Rey A. L'examen clinique en psychologie. Paris, France: Presses Universitaires de France; 1958.

E15. Lindeboom J, Schmand B, Tulner L, et al. Visual association test to detect early dementia of the Alzheimer type. J Neurol Neurosurg Psychiatry, 2002; 73(2): 126-33.

E16. Royall DR, Cordes JA, Polk M. CLOX: an executive clock drawing task. J Neurol Neurosurg Psychiatry 1998; 64: 588-94.

E17. Beck AT, Ward CH, Mendelson M, Mock J and Erbaugh J. An inventory for measuring depression. Arch Gen Psychiatry, 1961; 4: 561-71.
